# Complete Vision Recovery After Filler-Induced Blindness Using Hyperbaric Oxygen Therapy: Case Report and Literature Review

**DOI:** 10.1093/asjof/ojae036

**Published:** 2024-05-15

**Authors:** Rebecca Friedman, Allison V Coombs, Shanlee Stevens, Richard D Lisman, Ernest S Chiu

## Abstract

Injecting soft-tissue fillers, such as hyaluronic acid, has become an extremely popular method of facial augmentation. Although rare, adverse effects, ranging from cosmetically dissatisfactory to dangerous, may occur. The most severe adverse effect of these is vascular occlusion of the central retinal artery, resulting in vision loss. Protocols for the treatment of filler-induced blindness have not been well established, but there is evidence to suggest that hyperbaric oxygen therapy (HBOT) may aid in the therapeutic algorithm for filler-induced blindness. We present a clinical case of filler-induced blindness successfully treated with prompt administration of HBOT. A 38-year-old healthy female presented to the emergency room after immediate pain and complete vision loss following an at-home injection of mail-order filler into the left glabella and medial eyebrow. After treatment with hyaluronidase and ocular massage, neither of which relieved her symptoms, she received HBOT within 10 h of the injury, after which her vision improved significantly. After 2 additional sessions, the patient had complete vision recovery. This case report contributes to the very sparse literature documenting successful treatment of filler-induced blindness using HBOT, advocating for further study, and possible incorporation into the treatment algorithm for filler-induced blindness. Improper soft-tissue filler administration possesses a potential risk of severe adverse effects. It is crucial that the medical community is aware of treatments that offer the highest chance of visual recovery and sustained benefit for patients.

**Level of Evidence: 5:**

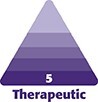

In recent years, injecting soft-tissue fillers, such as hyaluronic acid (HA), has become an extremely popular method of enhancing facial rejuvenation, wrinkle reduction, facial contour improvement, and facial volume augmentation. In 2022, over 23.7 million people received minimally invasive cosmetic procedures, and nearly 5 million people used HA fillers specifically.^1^ HA fillers consist of extracted and reformulated HA, a naturally occurring substance found in high concentrations in soft connective tissue and periorbital fluid.^2^ Because of their water-retaining glycosaminoglycans, they are especially useful for providing volume and reducing the appearance of depressions, fine lines, furrows, and scars, and have become one of the most popular choices of injectable filler.^2^ Although rare, adverse effects, ranging from cosmetically dissatisfactory to dangerous, may occur with injection. Recent studies indicate an adverse effect rate of 1 in 5000 to 10,000 injections.^3^

Filler-induced blindness is a devastating unintended outcome, resulting from vascular occlusion of the central retinal artery.^4^ This occurs more rarely; approximately 170 cases have been reported globally to date.^5^ The most common injection locations causing visual changes are the temporal, nasal, and glabellar regions.^4^ A recent review reported that only 20% of patients recovered vision after filler-induced blindness, whereas 15% saw some visual improvement and roughly 50% experienced complete and permanent vision loss.^4,6^ A previous review demonstrated complete visual recovery in only 2% of patients; this improvement to 20% may be because of increased preparedness, education, and early intervention in patients and injectors.^6^ Treatment protocols for filler-induced blindness are still not well established but typically include a combination of ocular physical maneuvers, hyaluronidase, intravenous steroids, ocular hypotensors, and supplementary oxygen.^6^ The diversity of medical specialists performing cosmetic facial procedures and the lack of high-quality evidence on treatment modalities contribute to this variety of treatment protocols.^7^ However, there is evidence to suggest that hyperbaric oxygen therapy (HBOT) may aid in the therapeutic algorithm for filler-induced blindness.^7,8^ We present a case of filler-induced blindness successfully treated with prompt administration of HBOT.

All procedures followed were in accordance with the ethical standards of the responsible committee on human experimentation (institutional and national) and with the Helsinki Declaration of 1975 (in its most recently amended version). Informed consent was obtained from the patient.

## CASE PRESENTATION

A 38-year-old female with no medical or ophthalmic history presented to the emergency room with acute vision loss in her left eye following an at-home injection of 0.5 cc mail-order HA filler (Multi-Crosslinking Hyaluronic Acid Glamour Filler, Fillers Korea, Seoul, South Korea) into her left glabella and left medial eyebrow. The patient's friend, who has no medical experience or qualification, injected the filler at 10:30 pm. She immediately noted severe pain and complete vision loss in her left eye, starting as a central blind spot that enlarged to engulf her entire visual field.

**Figure 1.**
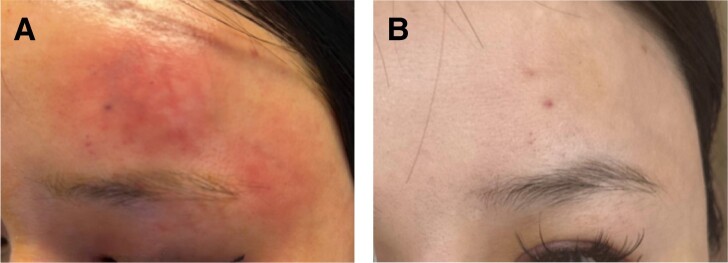
A 28-year-old female patient, upper left forehead. (A) Left periorbital region with external edema, erythema, skin mottling, and discoloration of the lid extending to the lash line within the V1 distribution. (B) Patient after 3 hyperbaric oxygen therapy treatments, 1 week postinjury.

**Figure 2.**
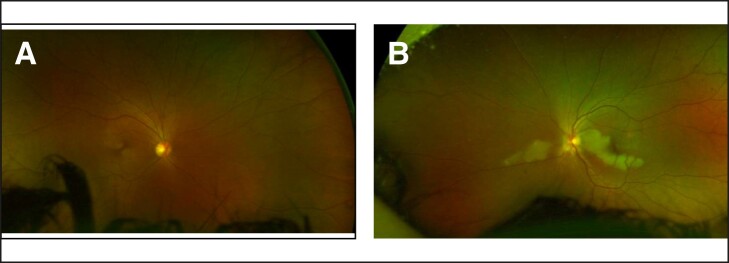
(A) Fundoscopic image of a normal right eye with normal optic disc, vasculature, and retinal appearance. (B) Affected left eye demonstrating dense white opacities of pallor, indicating retinal ischemia.

The patient was seen and examined at 1 am. The right eye examination demonstrated visual acuity of 20/20, whereas the left eye was hand-motion vision centrally and 20/400, eccentric in the far periphery. Bilateral intraocular pressure was 12 mm Hg with full extraocular motility. No afferent pupillary defect was noted in either eye. Externally, the left eye was noted to have edema and erythema, and skin mottling above the left eyebrow, as well as mottling and discoloration of the lid extending to the lash line within the V1 distribution (Figure 1). Other pertinent ophthalmic examination features included the left retina, which had a well-defined area of retinal pallor extending from the disc to the inferior macula. There were no visible emboli noted (Figure 2).

A diagnosis of early central retinal artery occlusion in the setting of filler injection was most likely, given the pattern of vision loss. An infiltration of 150 units of hyaluronidase was given at 1:30 am, 3 h after injury, with an additional 150 units 10 min later. Both were infiltrated into her supraorbital notch and overlying glabellar area in the V1 distribution and massaged to ensure proper distribution and penetration. The patient refused a retrobulbar hyaluronidase injection. She was given brimonidine-timolol to help lower intraocular pressure, and an ocular massage as is recommended for central retinal artery occlusions.

At 4:30 am, an additional 150 units of hyaluronidase were injected with massage. The patient had her first 90 min session of HBOT at 2 ATM, completed within 10 h of her injury. Her visual acuity was measured following the first HBOT session and was found to be 20/25 in the left eye, and her central scotoma had cleared so that she was left with only peripheral vision loss.

**Figure 3.**
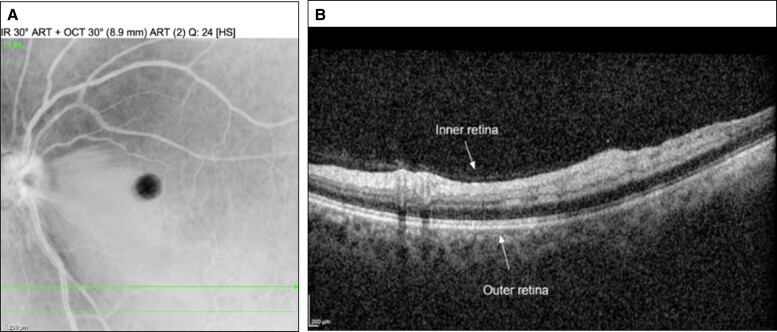
(A) En face and (B) raster spectral-domain optical coherence tomography of the left macula, revealing edematous and irregular inner retinal layers, representing an ischemic infarct of the inner retina.

**Figure 4.**
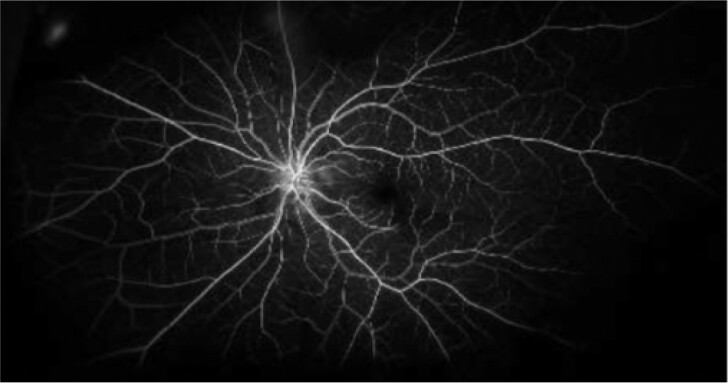
Fluorescein angiogram of the left eye without significant filling defect or areas of ischemia following treatment, 1 week postinjury.

After 3 HBOT sessions, vision recovered to 20/20. The patient did not complete the remainder of the recommended 10 sessions. Five days after injury, imaging of her left macula showed retinal ischemia (Figure 3), which was corroborated by optical coherence tomography. A fluorescein angiogram of the nasal macula demonstrated no significant filling defect or areas of ischemia (Figure 4).

## DISCUSSION

As injectable fillers increase in popularity, their use has become more widespread, including by nonmedical professionals. Increasing availability of direct-to-consumer filler possesses a high likelihood of severe adverse effects. Filler injections, while generally considered safe, carry a risk of adverse effects even when administered by experienced physicians.^7^ The increased prevalence of administration by nonmedical professionals who lack familiarity with facial anatomy or the risks and treatments of adverse reactions is necessary to be aware of.

There are very few reported cases in the literature of HA-induced blindness successfully resolved by a combination of hyaluronidase, ocular massage, and HBOT.^6^ Hyaluronidase is one commonly used component, but this is not always successful in reducing visual symptoms.^6^ It is thought that retrobulbar application of hyaluronidase may be effective in bringing the hyaluronidase closer to the area of blockage; however, it is controversial whether this method is effective.^6^ This patient refused retrobulbar hyaluronidase injection, and thus, this was not a factor in her recovery. HBOT has been gaining in popularity in the treatment of filler-induced blindness, as it has been shown to help improve visual outcomes as well as relieve the physical discomfort associated with this adverse event.^7^ Interestingly, this patient had only 3 sessions before she was back at baseline vision, whereas the typical protocol after filler-induced blindness includes 10 min or more of 90 min treatments daily. It is worth noting that the treatment is successful after just 3 sessions, which reduces the cost to the patient and increases compliance for the treatment period. The first session began within 10 h of injury, an extremely quick turnaround time for HBOT, which may have also contributed to treatment success. Although no formal vision testing was done between the application of HA and ocular massage and her first HBOT session, she did not report subjective improvement in vision until after her first HBOT session, and this change was reflected in her eye examination after that first session. From there, she continued to improve to baseline during the next 2 sessions and has since been at stable vision. Although it is not possible to determine the exact cause of her vision recovery, and it was likely a combination of multiple treatments, we believe that HBOT played a large role given her subjective improvement directly following HBOT without any improvement after multiple injections of hyaluronidase and massage.

A recent case series demonstrated HA-induced blindness treated with HBOT among other treatments; 2 had complete recovery of vision, 2 partially improved to light perception only, 1 had complete vision loss, and 1 patient's vision status was not reported.^9^ The patients who were started with HBOT most rapidly were those whose vision recovered.^9^ One case report of vision loss after forehead injection of HA resulted in permanent loss after 5 months; the authors indicated that HBOT had not been started within 24 h of injury which likely hindered its ability to alter the patient's prognosis.^10^ Four additional case reports of visual changes after HA injection treated with HBOT also showed no improvement; however, the exact protocol and timing of HBOT are not given.^11-14^ Notably, the immediate injection of hyaluronidase in a few of these cases did not improve visual symptoms.^9-11^ The reports of filler-induced blindness treated with HBOT and their outcomes are summarized in Table 1.

**Table 1.** Review of Cases of Filler-Induced Blindness Treated With HBOT

Case	Injection site	Symptoms	Signs	Treatment	HBOT given	Outcomes	Country
**1**	Nose	Left eye decreased vision, pain, nausea	Left eye no light perception, ptosis, ophthalmoplegia, MRI acute infarction	Hyaluronidase, low-level laser therapy, anterior chamber paracentesis, methylprednisolone, antiplatelet drugs, oral antibiotic, topical steroid, antibiotic eye drops, HBOT	6 h	Left eye vision light perception only, microphthalmia, resolved ophthalmoplegia, and ptosis	Thailand
**2**	Nose	Right eye vision loss, pain	Right eye no light perception, ptosis, ophthalmoplegia, RAPD	Hyaluronidase, ocular massage, breathing into plastic bag, carbogen, oral acetazolamide, dorzolamide/timolol, aspirin, HBOT	5 h	Right eye vision loss, resolved ophthalmoplegia, and ptosis	Thailand
**3**	Nose	Right eye blurry vision, periorbital pain, headache	Right eye visual field defect, erythematous patch	Hyaluronidase, nitroglycerin transdermal pad on chest, ocular massage, rebreathing into a plastic bag, subsequent pulse electromagnetic frequency, HBOT	4 h	Recovery of visual field defect	Thailand
**4**	Nose	Right eye vision loss, right eye periorbital pain, headache, nausea, vomiting	Right eye vision no light perception, ophthalmoplegia, ptosis, skin discoloration	Hyaluronidase, IV parecoxib, metoclopramide, acetazolamide, carbogen, timolol drops, aspirin, HBOT	12 h	Resolved ophthalmoplegia and ptosis, unclear vision status	Thailand
**5**	Forehead	Left eye decreased vision, headache	Left eye vision light perception only, ptosis, ophthalmoplegia, pupil dilation, superficial discoloration, small subacute infarction of left temporal lobe	Hyaluronidase, nitroglycerin pad on chest, ocular massage, rebreathing in plastic bag, IV antibiotic, systemic steroid, HBOT	Not stated	Left eye vision light perception only, partial improvement of ophthalmoplegia and ptosis	Thailand
**6**	Left temple	Left eye blurred vision	Blurred vision on examination, left eye ptosis	Hyaluronidase, ocular massage, HBOT	Within hours	Left eye vision recovery	Thailand
**7**	Forehead	Right eye vision loss, ocular pain	Right eye no light perception, ptosis, purple discoloration over nose, forehead	Hyaluronidase, aspirin, aspirin, oral acetazolamide, IV dexamethasone, HBOT	After 7 h, exact time not stated	Right eye vision of hand movements only	China
**8**	Not reported	Right eye vision loss, pain	Right eye no light perception	Hyaluronidase, oral acetazolamide, Ginkgo biloba extract, cobamamide, dexamethasone, HBOT	Not stated	Right eye vision no light perception	China
**9**	Forehead	Right eye vision loss	Right eye no light perception, pupil fixed, dilated, nonreactive, mottled erythema around injection site	Hyaluronidase, ocular massage, HBOT	Not stated	Right eye vision loss	China
**11**	Forehead	Right eye vision loss, retrobulbar pain	Right eye no light perception, no light reflex	Hyaluronidase, ocular massage, mannitol, high-flow O_2_, brimonidine, acetazolamide, aspirin, methylprednisolone, HBOT	Not stated	Right eye vision no light perception	Taiwan
**12**	Glabella	Right eye vision loss, nausea, pain, paralysis of limbs	Right eye no light perception, pupil dilated	Hyaluronidase, urokinase, HBOT	Not stated	Right eye vision loss	Japan

HBOT, hyperbaric oxygen therapy; RAPD, relative afferent pupillary defect.

There are still no clear recommendations on HBOT use in filler-induced blindness, and more research is needed in this area. Currently, it is not fully understood what helps restore vision in this setting, but it is thought that HBOT helps preserve oxygen flow to the inner retinal layers to prevent infarction of the ischemic area. The inner layers of the retina are perfused by the central retinal artery, whereas the outer layers are superficially perfused by the choroid through the posterior retinal artery and retain blood flow even when the central retinal artery is occluded.^15^ A greatly increased oxygen content of blood in a pressurized environment increases oxygen delivery to tissue up to 20-fold because of the increased partial pressure of oxygen.^16^ This bathes the ischemic area in oxygen to prevent infarction and irreversible damage of the tissue.^16^ The goal is to maintain adequate oxygenation until the embolus has resolved and/or collaterals have been established to the area, which can occur in as little as 24 h but typically takes 72 h or more.^17^ HBOT induces neovascularization to injured areas, which helps patients with ischemia establish those collaterals around the occluded vessel.^8^ This increase in microcirculation also helps reduce edema in the damaged area.^18^ HBOT is only effective on ischemic tissue that is not yet infarcted or necrotic, which may explain why vision did not improve in cases where HBOT delivery was delayed.^19^ The time it takes for tissue to transition from ischemia to infarct depends on multiple factors and differs across individuals, which further highlights the urgency of beginning HBOT as quickly as possible after injury.^19^

## Conclusions

This case report contributes to the very sparse literature, documenting the successful treatment of filler-induced blindness using HBOT, advocating for further study and possible incorporation of HBOT into the treatment algorithm for filler-induced blindness. This is the shortest number of treatments for HBOT reported, making it a more feasible and cost-effective modality for many patients. Additional studies are warranted to define best practice guidelines for this potentially devastating soft-tissue filler complication.
